# Remote stimulation of mitochondria and the antioxidant potential of thymocytes by weak non-ionizing non-thermal broadband pulsed electromagnetic fields in mouse

**DOI:** 10.3389/fphys.2026.1798354

**Published:** 2026-04-16

**Authors:** Denis Viktorovich Ivanov, Elena Vladimirovna Bondarchuk, Igor Feodorovich Turkanov, Irina Victorovna Kuzmina, Grigory Arnoldovich Flaks, Ekaterina A. Galkina, Valery Gryaznov

**Affiliations:** 1 Institute of Biomedical Research, Vladikavkaz Scientific Center of the Russian Academy of Sciences, Vladikavkaz, Russia; 2 Concern GRANIT JSC, Moscow, Russia

**Keywords:** antioxidant potential, biostimulation, electromagnetic fields, EMF, mice, mitochondria stimulation, mitochondrial fluorescence, mouse

## Abstract

Leading medical centers are currently considering pulsed electromagnetic therapy as a promising approach to prolong active life.

**Objective:**

The aim of this study was to investigate the effects of non-ionizing electromagnetic fields on mitochondrial activity and the state of the lipid peroxidation system, an antioxidant protection system in mice.

**Methodology:**

The studies were carried out on 60 BALB/c mice in accordance with bioethical standards and general ethical principles of animal experimentation. The animals were divided into 5 groups. The groups were placed in different rooms, standing at a considerable distance from each other. Experimental animals were exposed to non-invasive electromagnetic therapy device “TOR”. The mitochondrial activity of thymocytes and the total antioxidant potential of the blood plasma were analyzed in all animals.

**Results:**

The study showed that weak non-ionizing non-thermal broadband pulsed electromagnetic fields (EMF) increased the antioxidant potential of mouse blood: at minimum exposure (1 min/h/10 nights) by almost 8% (p > 0.05), at maximum (5 min/h/10 nights) - by 20.6% (p< 0.05) - compared to the negative control group (young animals not exposed to EMF). The experiment confirms the dose-dependent stimulating effect of EMF on the mitochondria of thymocytes; at maximum exposure, the changes become statistically significant (increase in mitochondrial fluorescence by 29% compared to the negative control group, p< 0.05).

**Conclusion:**

The biological effects of non-ionizing electromagnetic fields are diverse. Due to their high penetrating power, they are able to affect deep-seated organs and tissues, thereby opening up new approaches in medicine and regenerative therapy that are cost-effective for patients and elicit cellular responses similar to physical exercise.

## Introduction

There is now ample evidence that mitochondrial activity, which is involved in various types of metabolism - electron transfer in the respiratory chain cycle, carbohydrate, amino acid, and fatty acid metabolism - decreases with age, confirming the role of mitochondria in the aging process (Kotov et al., 2010).

It is known that one of the main functions of mitochondria is the regulation of synaptic release of acetylcholine. It had been shown that mitochondrial dysfunction leads to a decrease in its release (Lykhmus et al., 2014). At the same time, when the functional activity of mitochondria is impaired, there is a significant increase in the generation of reactive oxygen species (ROS), toxic byproducts of respiration, the excess of which causes the oxidation of unsaturated fatty acids, proteins and DNA. Mitochondrial DNA (mtDNA) has been found to be particularly sensitive to oxidative stress for a number of reasons, including the absence of protective histone-like proteins, low ability to repair damage, and proximity to the respiratory chain in the inner mitochondrial membrane (Halliwell, 2006). At the same time, each subsequent mtDNA lesion can increase oxidative stress by encoding the missing critically respiratory chain proteins, leading to further ROS generation and exacerbating mitochondrial oxidative damage (Evans et al., 2004). Oxidative mtDNA damage has been found to contribute to mitochondrial dysfunction in various mitochondria-related diseases, including aging, neurodegenerative diseases, and ischemia (Lin and Beal, 2006).

*In vivo* studies had been shown that radiofrequency electromagnetic fields (EMF) can lead to an increase in ROS production in cells after exposure (Consales, 2012). At the same time, studies are emerging that explore EMF as a promising therapeutic method capable of stimulating proliferative processes. The main effects of EMF may be a shift in the balance toward prooxidant processes, which, in turn, can trigger apoptosis-induced cell division to stimulate tissue growth (Hack et al., 2021). The biological effects of non-ionizing non-thermal EMFs are many-sided. Due to its high penetrating power, it can affect deep-located organs and tissues, thereby opening the door to new approaches in medicine. However, much research has focused on short-term effects, while many fundamental parameters remain unexplored (Kıvrak et al., 2017; Liang et al., 2023). Specifically, for weak non-ionizing non-thermal EMFs, issues related to mitochondrial activity remain largely unresolved, since complex mechanisms mediated through cellular oxidative stress may be involved.

There is a hypothesis about the effect of EMFs on mitochondrial respiratory complexes, which may be based on a model of biophysical interaction and takes into account the effect of these fields on the electrical and magnetic characteristics of biological membranes and molecules (Sharpe et al., 2021).

It is known that one of the possible mechanisms is the change in the conformation of the proteins of the respiratory complexes under the influence of EMFs. This can occur due to the dipole moments of the molecules and their orientation in the electric field, which in turn affects the interaction between the components of the respiratory chain and their activity (Luppo, 2026).

It has been shown that EMFs can affect ion channels in mitochondrial membranes, altering the permeability and transport of ions necessary for the functioning of respiratory complexes, which can lead to changes in the rate of oxidative phosphorylation and ATP production (Urbani et al., 2021). In addition, EMFs can interact with free radicals and antioxidant systems in mitochondria, affecting redox processes and membrane stability (Kıvrak et al., 2017).

Therefore, the aim of this research was to investigate the effects of weak pulsed EMFs on mitochondrial activity and the state of the «lipid peroxidation-antioxidant defense system» in laboratory animals.

## Materials and methods

The study was carried out on 60 male BALB/c mice purchased from the Andreevka branch of the Scientific Center for Biomedical Technologies of the Federal Medical and Biological Agency (Russia). After arrival at the vivarium, the animals were quarantined for 14 days. Housing conditions: 12:12 light control, temperature 22-24 °C, humidity and ventilation – according to the GOST 33215-2014 standard. In all experiments, with the exception of the positive control group #2 (old mice), young animals weighing 18-20 g aged 8-10 weeks were used. Old mice were 48-50 weeks old at the end of the study. All studies were performed in accordance with the ethical standards of the European Convention for the Protection of Vertebrate Animals used for Experimental and other Scientific Purposes (Strasbourg, March 18, 1986).

The animals were divided into 5 groups: group #1: negative control- 15 heads (young mice, 2 months); group #2: positive control (old mice, 12 months) - 10 heads; group #3: positive control 2 (oxidative stress induced by H_2_O_2_ by intraperitoneal administration of 100 μl of 1.5% H_2_O_2_ for 6 days once a day, young mice, 2 months) - 10 heads; group #4: EMF treatment (young mice 2 months, 1 min/h 10 days - 12 hours a night) - 10 heads; group #5: EMF treatment (young mice, 2 months, 5 min/h 10 days - 12 hours a night) - 10 heads.

For the treatment of animals in groups #4 and #5, the non-invasive electromagnetic therapeutic device “TOR” was selected in accordance with VEMP.941523.001TU, registered as a medical device under number RZN 2021/15459 in the Federal Service for Surveillance in Healthcare of the Ministry of Health of the Russian Federation on September 23, 2021. The operating principle of the medical device is based on the continuous remote effect of weak EMFs on a living organism (Fatenkov et al., 2024). “TOR” device treatment principle based on weak non-ionizing non-thermal EMFs continuously generated by high-voltage pulses with an amplitude of 5–8 kV, The pulse frequency was 100–150 Hz. Each wave packet with steep rectangular edges (meanders) contained frequency modes 25 kHz-fold. The operating power of the device was about 12 Watt (maximum power in hot atmospheric conditions does not exceed 150 Watt) (AKTCIONERNOE OBSHCHESTVO "KONCERN GRANIT", 2022).

At the distance from the device emitter 10 cm the magnetic strength of pulsed magnetic field - 0,13 µT and electric field strength 38V/m (50Hz); measured radiated power density e.g. for EMF spectra 500MHz, 2,45GHz - 38 and 36µW/cm2, respectively, and distance 20cm decayed- 2,1 and 1,6µW/cm2, correspondingly.

No adverse events were detected of this technology are not discovered to date.

In group #4, the device operated in pulsed mode for 10 nights with a treatment mode of 1 min/h, followed by the development of the effect, mice were euthanized after 30 days. The EMF treatment of group #5 was carried out for 10 nights in a 5min/h mode, and then according the same procedure as for group #4. Two total exposures during 10 days were chosen during 10 days in this experiment: 2 hours (1 min*12 times a day) and 10 hours (5 min*12 times a day), based on experience of previous studies on enhanced healing of rats with fractured bones by weak EMFs (Kuzmina et al., 2025) with a total exposure duration for 3.5 hours per the therapy period 14 days (5 min* 3 times a day). The device was positioned in the same plane with the treated mice at a distance of 5 m.

The presence of the vendor representatives during the arranging the experimental and control groups of animals ensured the reproducibility of the results and the reliability of the strict condition that the EMF of the “TOR” device would in any way affect the control group, and that there would be no electromagnetic influences from external sources that could significantly change the geomagnetic background in the cages with animals.

At the end of the experiment, the animals were euthanized with cervical dislocation after preliminary anesthesia with isoflurane. Then, *ex tempore*, blood was collected from the heart using insulin syringes, stabilized with heparin (at a final concentration of 50 IU/ml). The mice were then dissected and their thymus was removed for analysis.

*Mitochondrial activity of thymocytes* was determined using commercial MitoTracker – Tetramethylrhodamine kits (Thermo Fisher Scientific, USA). For this, thymus samples were ground at 20 °C in a glass homogenizer in 7 ml of 3.0% human albumin prepared in medium 199 supplemented with Earle’s salts and glutamine (PanEco, Russia). The resulting cell suspension was filtered through two layers of nylon and centrifuged for 5 minutes at 400 G, the supernatant was removed, 3.0% albumin prepared in phosphate buffer with pH 7.4 (PB) was added to the sediment, and the cells were resuspended. The cells were then incubated for 10 minutes at 37 ± 0.2 °C. A 250 μl aliquot was taken from the cell suspension and stained with MitoTracker solution at the same temperature for 25 minutes. The resulting mixture was then diluted 1:1 with a 3.0% albumin solution in PB and centrifuged for 5 minutes at 400 G. 5 μl of cell mass were collected from the sediment and smears were prepared on a V-Sampler hematology sampler (Austria), air-dried and analyzed using an Evos M7000 imaging system (Thermo Fisher Scientific, USA) in RFP mode at 400x magnification. 312 micrographs were taken for each mouse organ sample, containing an average of 50 cells in each image. The average cellular fluorescence (I, mean intensity) was determined and mitochondrial fluorescence (M.F.) was calculated using the formula.

(1) М.F. (a.u.) = I×SI/100

where SI is the average luminescence area; 100 is an empirical coefficient facilitating the analysis and interpretation of the results. The thymocyte cell mass was analyzed without differentiation into individual components, allowing us to express the total mitochondrial activity of the organs studied (Waters, 2009).

*The total antioxidant activity* of mouse blood plasma was determined using the generally accepted FRAP (Ferric Reducing Antioxidant Power) technique (Benzie and Strain, 1996). FRAP has certain limitations in determining the difference between enzymatic-non enzymatic antioxidants or ROS signaling- scavenging (Benzie and Devaki, 2017; Echegaray et al., 2021) For this purpose, heparin-stabilized blood was centrifuged at 3000 rpm for 10 minutes. Optical density was determined at a wavelength of 593 nm using a Shimadzu 1650 PC spectrophotometer with a reaction mixture incubation time of 5 minutes at room temperature. The results were expressed as total antioxidant potential (TAP):

(2) $$TAP=\;{OD}^{*}100$$

where OD (Optical Density) is the optical density (the difference between the experimental and control samples); 100 is a coefficient facilitating the data perception and interpretation (Aniqa et al., 2025).

Statistical analysis was performed by STATISTICA 10.0 software packages, calculating the median (Me) and upper and lower quartiles (C25-C75). The significance of the obtained results (p) was assessed using the Mann-Whitney U-test; results were considered statistically significant at p< 0.05.

## Results

**Figure 1:**
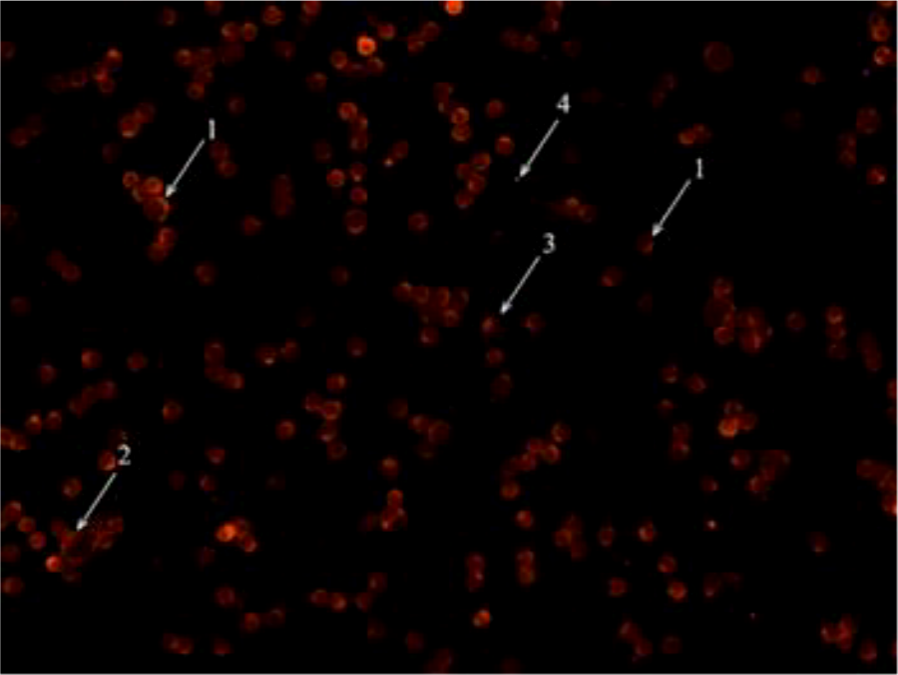
Micrograph of thymocytes with stained mitochondria: 1 - lymphocyte; 2 - lymphoblast; 3 - macrophage; 4 - platelet (scale 1:400).

**Figure 1** shows a typical micrograph of thymocytes with stained mitochondria.

**Figure 2:**
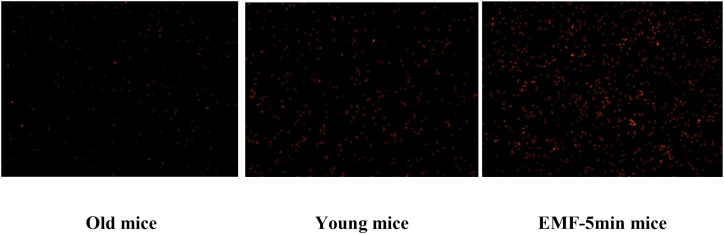
Micrographs of average cellular fluorescence for different groups of laboratory animals.

**Figure 2** shows that the EMF 5 min/h exposure (group #5) had a biostimulation effect on the mitochondria of mouse thymocytes (the brightest fluorescence is visible in micrographs).

**Figure 3:**
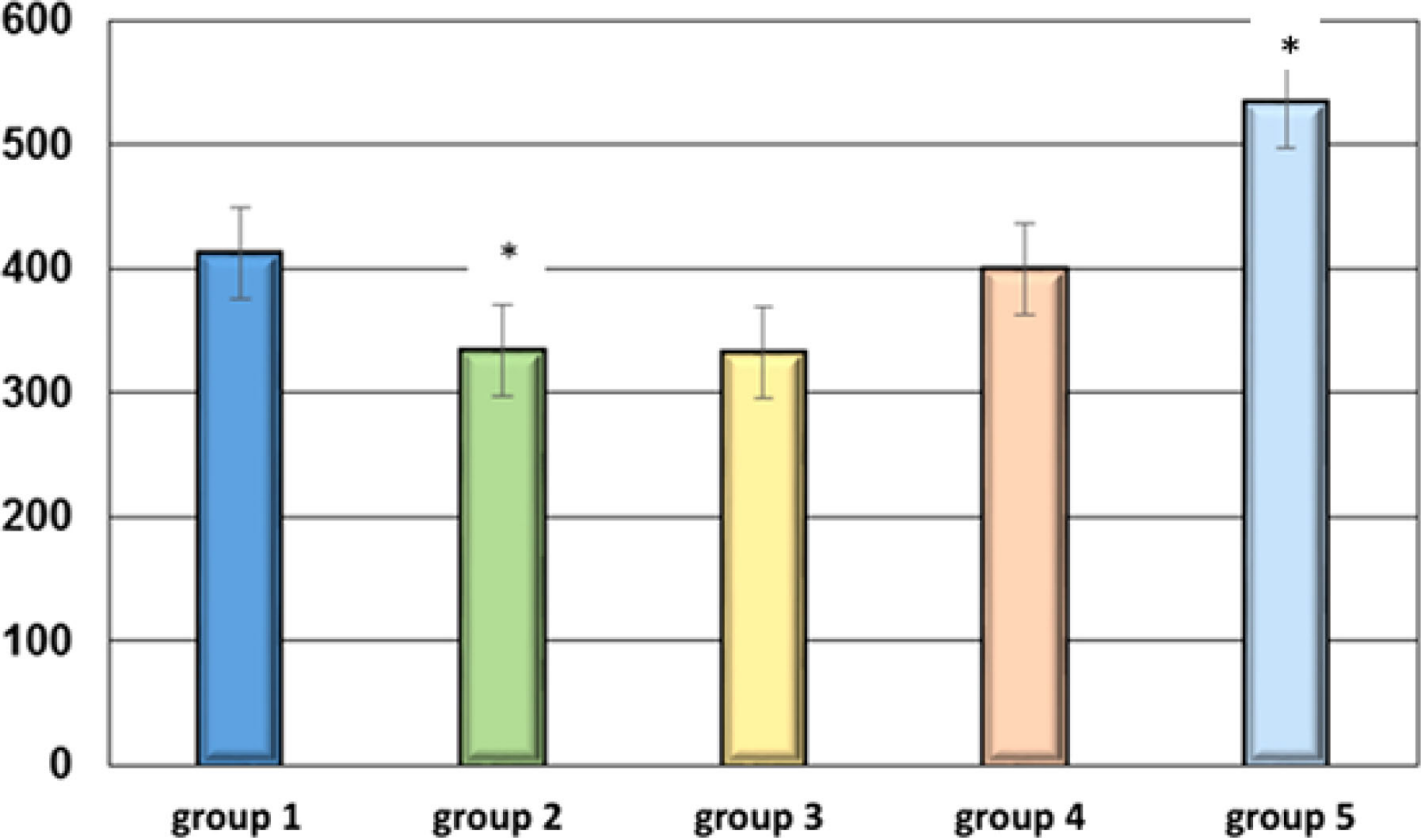
Results of assessing the effect of EMF on mitochondrial fluorescence (units) in BALB/C mice. * p<0.05 – relative to the negative control group.

**Figure 3** illustrates a significant increase in the metabolic activity of intracellular organelles in the group #5 exposed to 5min/h EMF mode by 29% (p<0.05) compared to the negative control group.

In group #4, mitochondrial activity values indicated the presence of a biological effect, which is manifested by high individual variability of the reaction, which, however, does not allow for statistically significant differences compared to animals in the control group. In the positive control group, a consistent decrease in mitochondrial metabolic activity was observed (p< 0.05), which is consistent with the data of the scientific literature (Das and Muniyappa, 2013), where a decrease in mitochondrial metabolic activity with age was emphasized.

**Figure 4:**
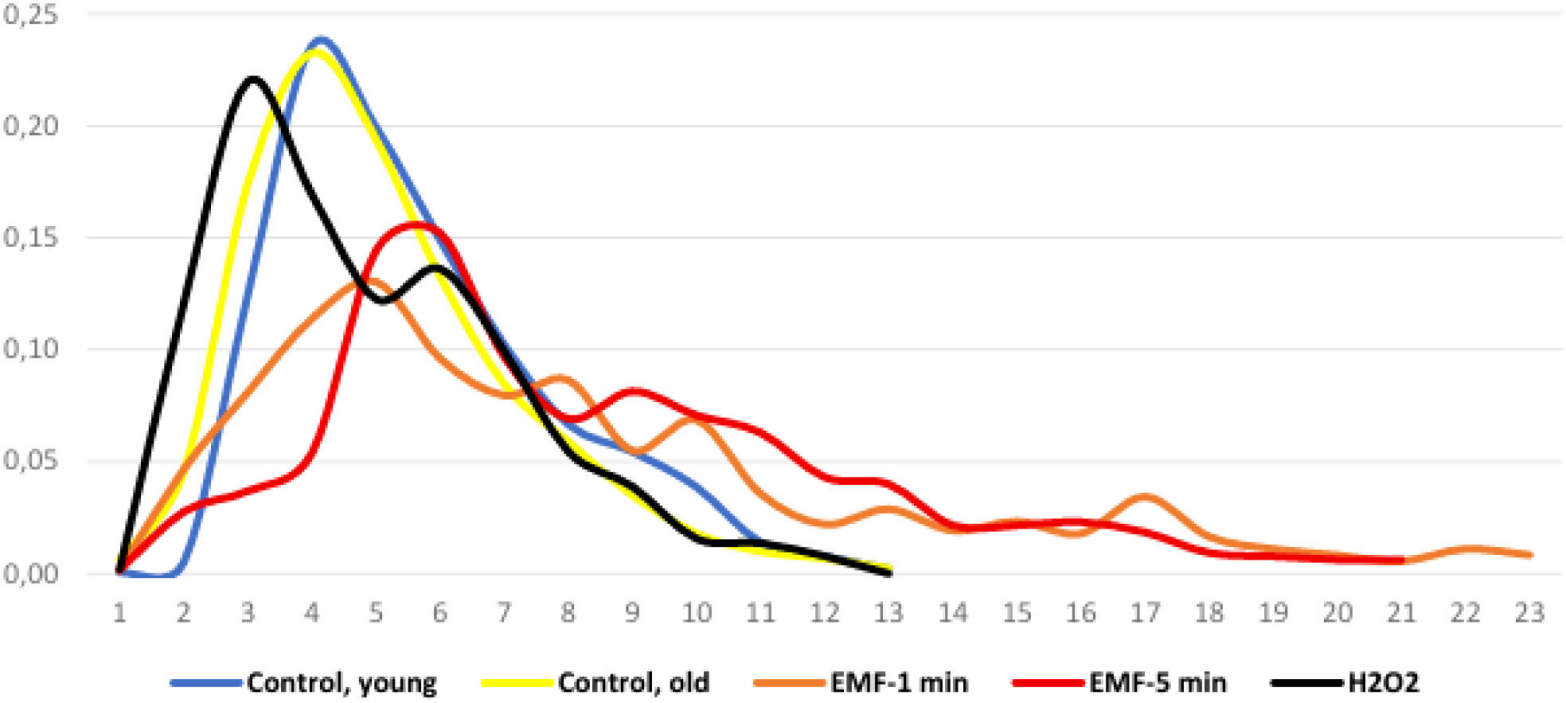
Fluorescence intensity distribution (Abscissus axis – fluorescence intensity, a.u.).

**Figure 4** plots fluorescence intensity graphs of studied groups, compiled from an analysis of the micrograph array (**Figure 2**). Analysis of these graphs reveals that the fluorescence intensity maxima for each EMF-treated group shift toward higher values, indicating increased mitochondrial fluorescence intensity.

The Pearson coefficients calculated based on fluorescence analysis data for all groups relative to group #1 (negative control, young mice) are presented in **Table 1**.

**Table 1:** The Pearson coefficients.

Group 1 (young mice) to group 2 (old mice)	Group 1 (young mice) to group 3 (H_2_O_2_)	Group 1 (young mice) to group 4 (EMF-1min)	Group 1 (young mice) to group 5 (EMF-5min)
0,98	0,86	0,91	0,57

This table shows that the correlation coefficient between group #1 and group #5 is significantly reduced compared to the other group pairs. This indicates that the mouse population, exposed to EMF for 5 minutes, experienced radical changes in mitochondrial activity compared to the young mice in group #1. However, according to Mann–Whitney U-test group #1 and group #2 are NOT significantly different, but group #1 and #3 (EMF-1min), group #1 and#5(EMF-5min) are significantly different with the confidence level 0, 05.

**Figure 5:**
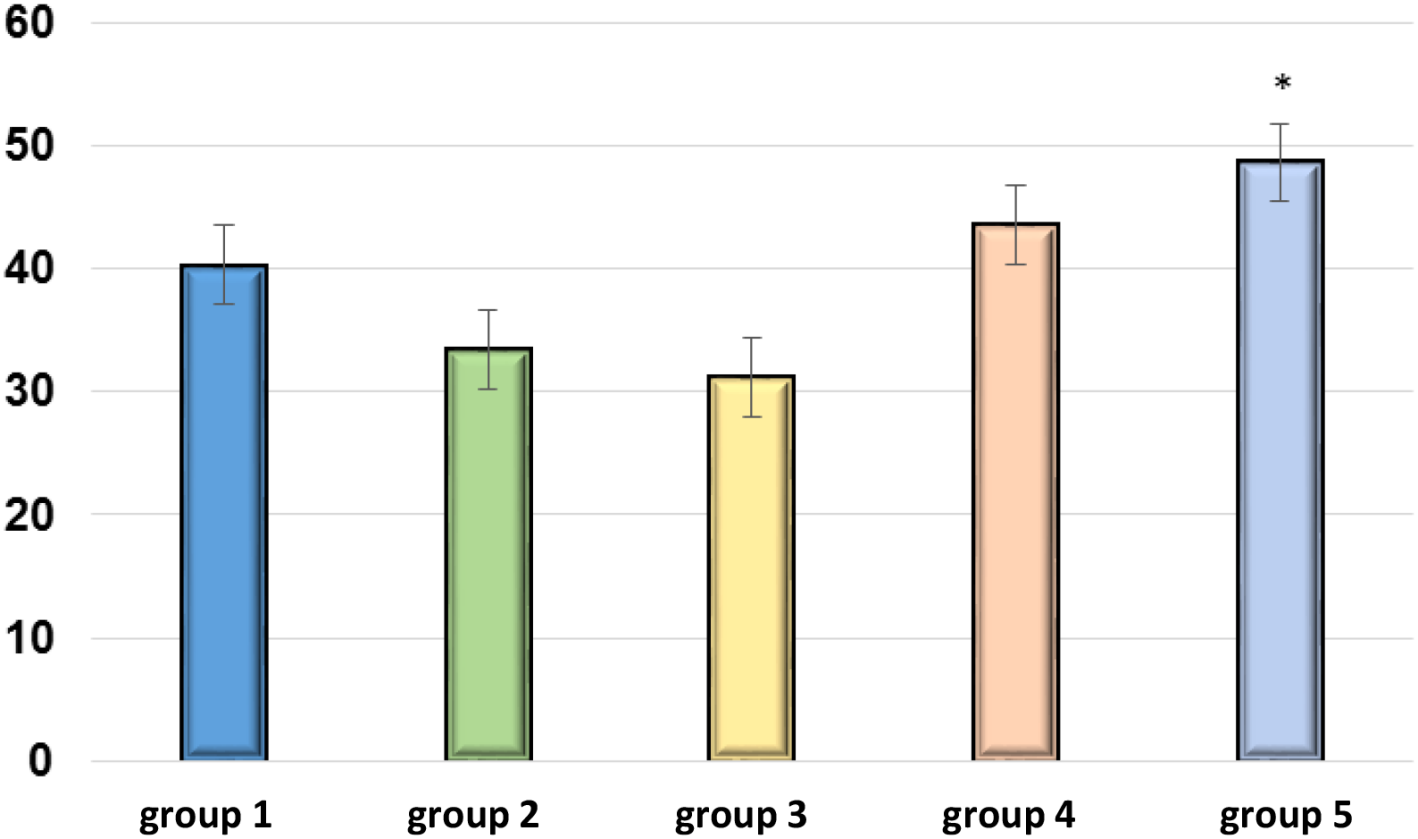
Total antioxidant potential of blood plasma of BALB/c mice (a.u.). * p<0.05 – relative to the negative control group.

The EMF effect on the total antioxidant potential of mouse blood, assessed by the ability to reduce Fe(III) to Fe(II), as shown in **Figure 5**.

**Figure 5** shows EMFs promoting the dose-dependent increase in the antioxidant potential of the blood. At the minimum exposure (group #4), it increased by 7.8% (statistically insignificant), and at the maximum exposure (group #5), it increased by 20.6% (p<0.05) relative to the negative control group.

## Discussion

Changes in mitochondrial functional activity play a key role in cellular adaptation to variations in oxidative metabolism. Modern research shows that changes in mitochondrial morphology are associated not only with their functional capacity, but also have a significant impact on cellular and overall metabolism. These changes can affect the cellular redox status, the regulation of apoptosis, signaling pathways, and the overall energy balance of the body (Hackenbrock, 1966; Buck et al., 2016).

In addition to their role in metabolism, evidence is accumulating for their role in a number of other functions: cell proliferation, cell death, migration, ROS production, and mitophagy. It is noteworthy that all of these processes are essential for the proper functioning of the adaptive immune system and thymus development (Sharpe et al., 2021).

A decrease in mitochondrial activity is believed to indicate cellular aging. During aerobic respiration, cells produce ROS, which contain an unpaired electron. These ROS, in turn can damage molecules within cells and impair their function. The accumulation of oxidative damage over time leads to a decline in tissue and organ function and, ultimately, aging. Currently, interest in cellular aging has largely focused on the role of senescent cells in the aging process. Senescent cells accumulate with age in a variety of tissues across a number of species, although the exact proportion of senescent cells in these tissues remains controversial due to the lack of a single aging biomarker. Mitochondria in senescent cells exhibit significant changes in activity and morphology, many of which are similar to those that lead to the pathologies of aging (Hruby and Higuchi-Sanabria, 2025).

There is currently a growing number of studies describing the positive effects of EMF on mitochondria (Hollenberg et al., 2021). Experimental data show that radiofrequency EMFs in the range of 3 to 5 MHz are capable to alter the modulation of mitochondrial signaling, affecting cell growth, mitochondrial mass, and oxidative stress (Gurhan and Barnes, 2023; Gurhan et al., 2023). In this regard, EMF may serve as a therapeutic tool for selectively modulating oxidative stress and mitochondrial function in cancer cells, as antioxidants play a key role in mitigating potential side effects (Gurhan and Barnes, 2024).

*In vivo* experiments on mouse tibial fracture models have shown that EMF treatment improves fracture healing by enhancing biomechanical properties and increasing callus mineralization. Overall, EMF promotes bone fracture healing by activating mitochondrial oxidative phosphorylation (Hollenberg et al., 2021; Akimoto et al., 2024). This may be caused by improved mitochondrial bioenergetics in the muscles, reduced systemic lipotoxicity, enhanced calcium signaling, and improved mitochondrial respiration when exposed to EMFs which is equivalent to physical exercises (Yap et al., 2019; Venugobal et al., 2023; Teranishi et al., 2024; Ito et al., 2025).

The results of this study demonstrate the significant potential of weak pulsed non-ionizing, non-thermal broadband electromagnetic fields to improve mitochondrial physiological activity. It is important to emphasize here that weak EMFs in the radio frequency range, as in the case of our EMF treatment, affect living organisms without heating, since the emitted radiophotons have significantly lower energy than 10^-3^ kT (Buchachenko, 2014).

It is hypothesized that external pulsed EMFs may modulate mitochondrial respiratory complexes, promoting the optimization of reactive oxygen species formation and antioxidant synthesis in these organelles. This, in turn, will contribute to slowing the aging process of the cell and the organism as a whole.

## Conclusion

The presented results demonstrate that remote dose-dependent exposure to EMF exerts a statistically significant effect on experimental groups of laboratory animals at an exposure time of 5 minutes per hour. This exposure results in a statistically significant increase in mitochondrial fluorescence and a 20.6% increase in the antioxidant potential of the blood. These changes may indicate a positive effect of EMF on metabolic processes in the animals and humans.

## Data Availability

The datasets presented in this study can be found in online repositories. The names of the repository/repositories and accession number(s) can be found in the article/supplementary material.
